# *Brassica*-Specific Orphan Gene *CROG1* Confers Clubroot Resistance in Arabidopsis via Phenylpropanoid Pathway Activation

**DOI:** 10.3390/plants14172683

**Published:** 2025-08-27

**Authors:** Jingyi Zheng, Yana Zhou, Yan Sun, Xiaonan Li

**Affiliations:** 1 College of Horticulture, Shenyang Agricultural University, Shenyang 110866, China; amberz0301@163.com (J.Z.); syauzyn@163.com (Y.Z.); 2 Dalian Modern Agriculture Development Service Center, Dalian 116012, China; syesy0707@163.com

**Keywords:** *Brassica rapa*, clubroot resistance, orphan gene, *Arabidopsis thaliana*

## Abstract

Clubroot disease, caused by *Plasmodiophora brassicae*, poses a serious threat to global *Brassica* crop production. Orphan genes (OGs), which are species or lineage-specific and lack detectable homologs in other taxa, have been implicated in various biotic stress responses. Here, we identified a novel *Brassica rapa*-specific orphan gene, designated *CROG1*, that confers resistance to clubroot. Heterologous overexpression of *CROG1* in *Arabidopsis thaliana* significantly enhanced resistance to *P. brassicae*. Transcriptomic profiling of *CROG1*-overexpressing lines highlighted the essential role of the phenylpropanoid biosynthesis pathway, showing upregulation of key lignin synthesis genes (including *CCoAMT, CAD6, PER4,* and *AZI1*) and defense-related regulators (*RBOHC* and *WAKs*). Weighted co-expression network analysis further corroborated the link between *CROG1*-mediated resistance and enhanced lignin deposition and cell wall reinforcement. Our findings establish *CROG1* as a *Brassica*-specific orphan gene that enhances clubroot resistance via phenylpropanoid pathway activation. These results highlight the potential of orphan genes as novel genetic resources for breeding clubroot-resistant *Brassica* varieties, offering a sustainable strategy to mitigate yield losses caused by this devastating disease.

## 1. Introduction

Clubroot, a soil-borne disease caused by *Plasmodiophora brassicae* Woronin, is a significant threat to *Brassica* crop production [1]. The life cycle of this pathogen comprises three stages: (1) survival as dormant spores in the soil; (2) primary infection of root hairs and epidermal cells; and (3) secondary infection of the cortex [2]. This infection process induces abnormal cell proliferation and expansion in infected tissues, leading to gall formation. *Plasmodiophora brassicae* resting spores can persist in soil for over 15 years, and the frequent mixed occurrence of physiological races in field populations is a challenge for disease control [3]. This complexity has increased the demand for disease resistance gene identification. Clubroot resistance genes and quantitative trait loci have been mapped in *Brassica* species, originating from A-genome crops, such as turnip (*Brassica rapa* ssp. *rapa*) and Chinese cabbage (*Brassica rapa* ssp. *pekinensis*). To date, 28 resistance genes/loci have been identified in the A genome, concentrated on chromosomes A01, A02, A03, A05, A06, and A08 [4]. Two major clusters of CR genes are present on A03: one cluster harboring three CR loci (*CRd*, *CRk*, and *Crr3*)*,* and the second cluster harboring seven CR loci (*CRa*, *CRb*, *CRq*, *Rcr1*, *Rcr2*, *Rcr4*, and *Rcr5*) [5]. Among these genes, *CRa* [6] and *Rcr1* [7] have been successfully cloned.

A critical defense strategy against *P. brassicae* involves the fortification of the plant cell wall, which serves as a primary physical and chemical barrier against pathogens [8]. Dynamic cell wall remodeling, including lignification and structural modifications, constitutes a fundamental component of the plant immune response [9,10,11]. Notably, genomic analyses reveal that *P. brassicae* possesses a limited repertoire of cell wall-degrading enzymes [12], suggesting that observed alterations in host cell walls during infection are likely driven primarily by the plant’s own defense mechanisms rather than direct pathogen-mediated degradation. Central to this defense-associated cell wall reinforcement is the phenylpropanoid pathway, a major secondary metabolic route responsible for the biosynthesis of lignin, flavonoid, and phenolic acids [13]. Lignin deposition directly enhances cell wall integrity [14], while flavonoids and phenolic acids function as antimicrobials, antioxidants, and signaling molecules [15]. Accumulating evidence underscores the pivotal role of the phenylpropanoid pathway, particularly lignin biosynthesis, in clubroot resistance across *Brassica* species. For instance, Tu et al. [16] demonstrated that clubroot resistance conferred by the *Rcr1* gene stems from its induction of lignin accumulation, supporting the contribution of basal defense activation and lignin synthesis via the phenylpropanoid pathway for clubroot resistance. Similarly, both flavonoid and lignin biosynthesis pathways are significantly induced in resistant plant material [17]. Irian et al. [18] demonstrated that infection by *P*. *brassicae* activates the expression of phenylpropanoid pathway-related genes in oilseed rape (*Brassica napus*). Disease-resistant lines exhibited upregulated expression of genes involved in lignin and flavonoid biosynthesis. Ergothioneine (EGT) enhances clubroot resistance by inducing the expression of lignin synthesis-related genes in Chinese cabbage [19]. The critical role of cell wall synthesis and phenylpropanoid-associated lignin synthesis has been corroborated by multiple omics studies. Transcriptome studies have consistently reported the altered expression of cell wall-related genes in *Brassica* crops infected by *P*. *brassicae* [20,21,22,23,24]. These findings have been further corroborated by metabolomic analyses across multiple plant species. The differential accumulation of key secondary metabolites, particularly flavonoids, lignans, and phenylpropanoid derivatives, has been documented in *A*. *thaliana*, *B*. *napus*, and *B*. *rapa* after pathogen infection [14,19,25,26]. Collectively, these findings establish the enhancement of the phenylpropanoid pathway—particularly lignin biosynthesis for cell wall reinforcement—as a central mechanism of clubroot resistance. However, the key regulatory genes orchestrating this defense-specific activation in Brassica species remain poorly characterized. This knowledge gap is particularly significant given the potential contribution of lineage-specific genes to host-pathogen coevolution and specialized defense responses.

Orphan genes have arisen through diverse pathways [27] and have been linked to environmental adaptation, growth, and development. These genes exhibit evolutionary lineage specificity [28]. *Brassica rapa* shows substantial phenotypic variation and diverse resistance responses to *P*. *brassicae*. The potential involvement of clubroot-resistant orphan genes in host adaptation to *P*. *brassicae* represents a compelling research direction. Observed correlations in abundance shifts suggest that orphan genes play a functional role in mediating the resistance responses of *B*. *rapa* to pathogen infection [29]. In our prior study, we conducted a functional screen of a *Brassica* orphan gene overexpression library to identify novel components of plant immunity [30]. Our systematic analysis revealed that transgenic *Arabidopsis* plants overexpressing *BrOG51* had significantly enhanced resistance to *Pseudomonas syringae* pv. *tomato* DC3000 (*Pst* DC3000) infection, as indicated by the reduction in bacterial proliferation and disease symptoms. These findings establish *BrOG51* as a previously uncharacterized genetic determinant of pathogen resistance, suggesting that orphan genes are an untapped reservoir for plant defense mechanisms.

Given the well-documented role of phenylpropanoid-mediated cell wall reinforcement in clubroot resistance, coupled with emerging evidence that orphan genes (OGs) may encode novel regulators of plant defense pathways, we hypothesized that *Brassica OGs* could play significant roles in conferring resistance to *P. brassicae*. To test this hypothesis, the present study screened a panel of *B. rapa* orphan gene (*BrOG*) overexpression lines in *Arabidopsis* for altered responses to *P. brassicae* infection; identified and characterized candidate resistance-conferring *OGs* through phenotypic, molecular, and transcriptomic analyses; and elucidated the molecular mechanisms by which candidate *OGs* enhance clubroot resistance, with particular emphasis on their potential role in regulating the phenylpropanoid biosynthesis pathway and associated cell wall reinforcement processes. Through this approach, we identified *BrOG51* (designated *CROG1*) as a novel *Brassica*-specific orphan gene involved in clubroot resistance and further investigated its functional mechanisms using comprehensive transcriptomic and molecular analyses.

## 2. Results

### 2.1. Characterization of Clubroot Resistance in BrOGs Overexpression Lines

**Figure 1:**
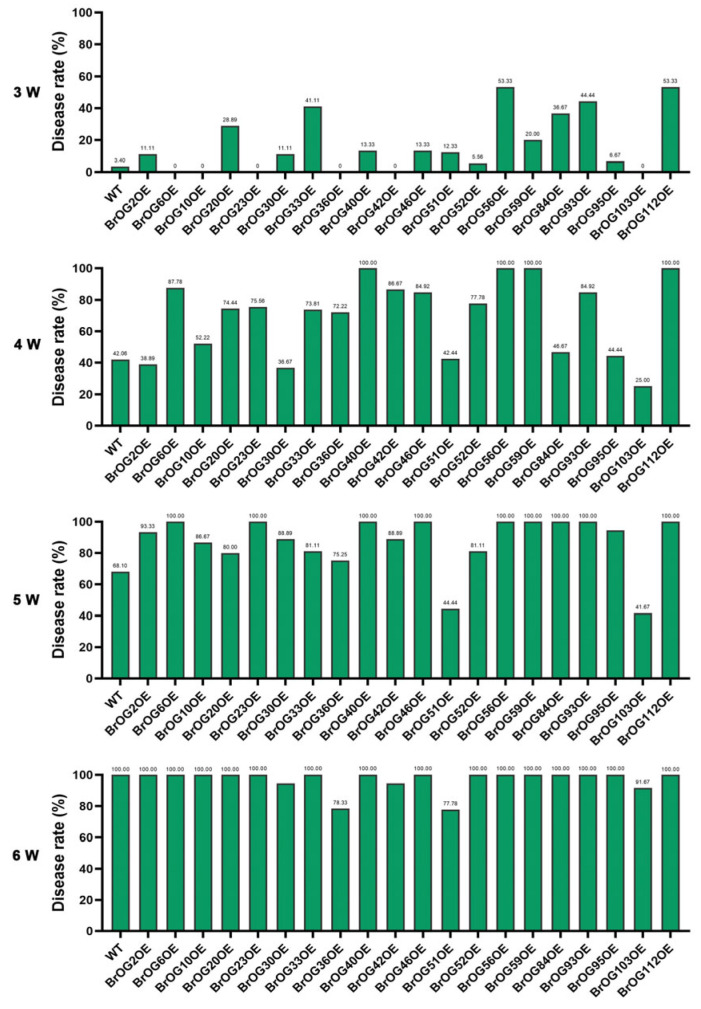
Disease rate (DR) of WT and 20 *BrOG*-overexpressing *Arabidopsis thaliana* transgenic lines observed at 3–6 wpi with *Plasmodiophora brassicae*. At least 36 plants per family line were surveyed per period; the DR values are indicated above the columns. WT represents *A. thaliana* ecotype Col-0.

**Figure 2:**
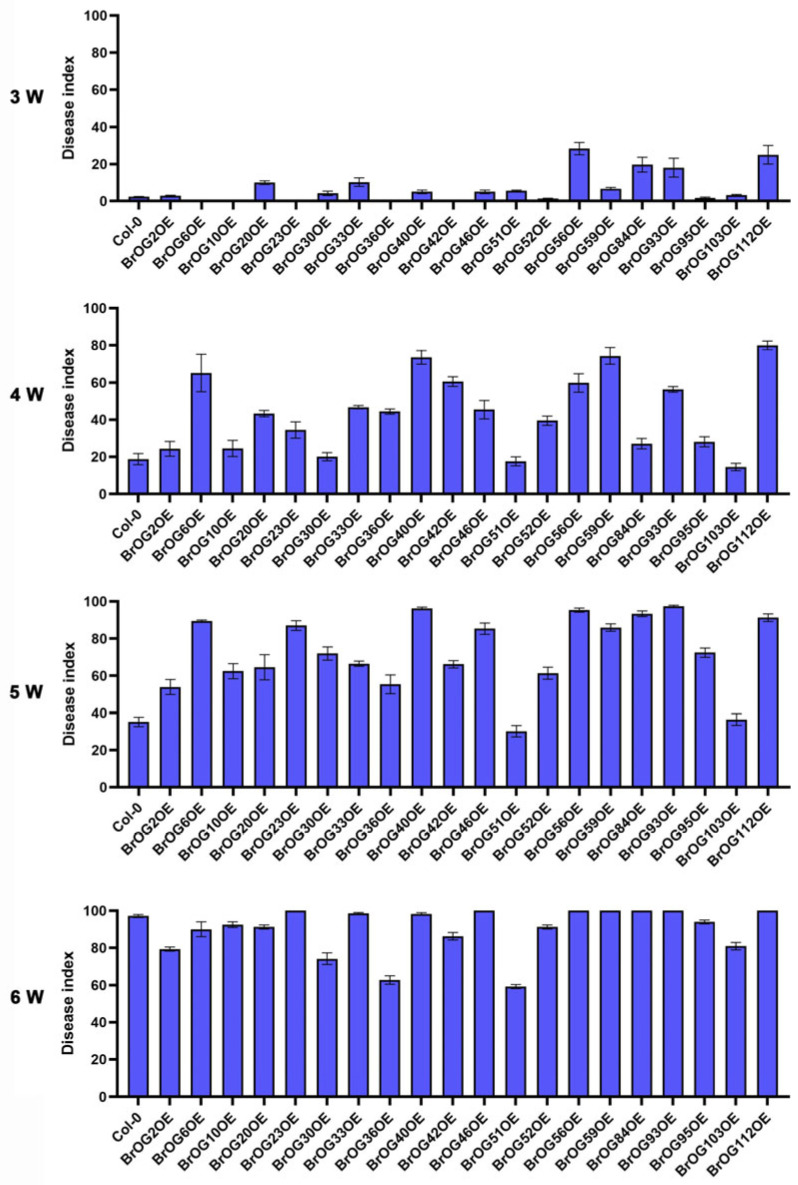
Disease index (DI) of WT and 20 *BrOG*-overexpressing *Arabidopsis thaliana* transgenic lines observed 3–6 wpi with *Plasmodiophora brassicae*. At least 12 plants per family line were surveyed per period with three biological replicates; the DI values are indicated above the columns. WT represents *A. thaliana* ecotype Col-0.

We screened 20 transgenic *A*. *thaliana* lines from our pre-established *BrOG* overexpression library in order to determine the roles of orphan genes in plant disease resistance, and [30] for resistance to *P*. *brassicae*. The disease rate (DR) and disease index (DI) were determined at 3, 4, 5 and 6 weeks post-*P*. *brassicae* inoculation (wpi). Three transgenic lines—BrOG36OE, BrOG51OE, and BrOG103OE—exhibited significantly reduced susceptibility compared with wild-type (WT) plants (Figure S1). At 6 wpi, these lines maintained lower disease rates (77.78–91.57%), but all of the WT plants showed 100% disease rates (Figure 1). Further, their disease index values (59.70–80.60) were substantially lower than that of WT (96.4) (Figure 2). BrOG51OE displayed the strongest resistance phenotype, suggesting that its orphan gene plays a critical role in pathogen defense.

### 2.2. BrOG51OE Showed Significant Resistance to Both Pseudomonas syringae pv. Tomato DC3000 and P. brassicae Infection

Inoculation of the three transgenic lines (BrOG36OE, BrOG51OE, and BrOG103OE) with *Pst* DC3000 resulted in enhanced disease resistance compared with the WT plants. Leaves of these transgenic lines showed less chlorosis (Figure S2A) and milder disease symptoms (Figure S2B) at 3 days post-inoculation (dpi). As *Pst* DC3000 triggers the salicylic acid (SA) signaling pathway in *A*. *thaliana*, we quantified the transcript levels of the SA-responsive marker gene *AtPR1*. *AtPR1* expression was significantly upregulated in all three transgenic lines (Figure S2C), especially BrOG51OE.

**Figure 3:**
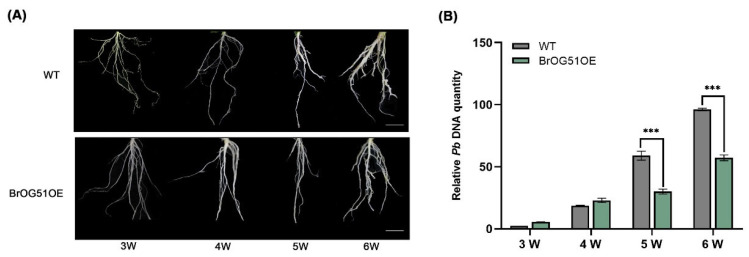
Disease investigation of transgenic line BrOG51OE at 3, 4, 5, and 6 weeks after inoculation with *Plasmodiophora brassicae*. (**A**) Root phenotypes of transgenic line BrOG51OE inoculated with *P*. *brassicae*. Scale bar = 2 cm. (**B**) Relative *P*. *brassicae* DNA content in *Arabidopsis thaliana* wild type (WT) and BrOG51OE determined at 3, 4, 5, and 6 weeks after inoculation with *P*. *brassicae*. The data are presented as means ± SE from three independent measurements. Black asterisks (***) indicate significant differences between treatments (*t*-test, *p* < 0.05).

BrOG51OE exhibited significant resistance to clubroot disease caused by *P*. *brassicae*. Its resistance persisted even at 6 wpi, contrasting with WT plants (Figure 1 and Figure 2), which were susceptible at this stage (Figure 3A). Consistent with the phenotypic observations, BrOG51OE plants showed a lower *P*. *brassicae* DNA content than WT controls (Figure 3B), particularly at 5 and 6 wpi, indicating effective suppression of pathogen proliferation. These findings collectively support the hypothesis that the orphan gene *BrOG51* contributes to clubroot resistance. Consequently, we designated this gene *CROG1* (*Clubroot-Related Orphan Gene 1*) to reflect its status as the first reported orphan gene involved in clubroot resistance.

### 2.3. Analysis of CROG1 Expression in Brassica rapa After Inoculation with P. brassicae

**Figure 4:**
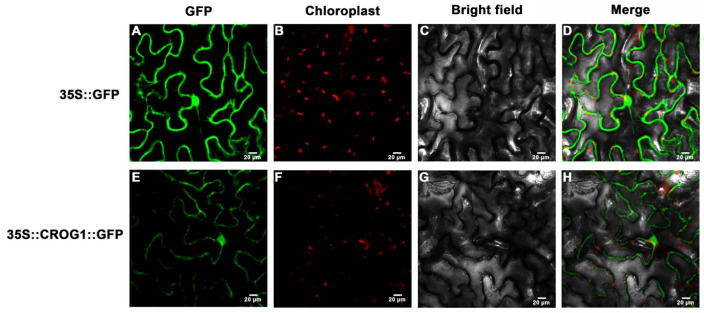
CROG1 subcellular localization analysis. (**A**,**E**) GFP fluorescence. (**B**,**F**) Chloroplast signals. (**C**,**G**) Bright field images. (**D**,**H**) Merged images. A Leica confocal microscope was used to collect images at 48 h following agro-infiltration. Control GFP localization was evident in these cells. Scale bar: 20 mm.

*CROG1* (*BrOG51*, *BraA05004304*), which was identified in the *B*. *rapa* reference genome v2.5 [30], is a *Brassica*-specific gene with no detectable homologs outside the *Brassica* genus. The gene spanned 737 bp, contained two exons and one intron, and encoded a predicted 49-amino-acid protein (Figure S3). Subcellular localization assays with a 35S:: CROG1::GFP fusion construct (versus 35S::GFP control) revealed the predominant localization of CROG1-GFP in the nucleus and cell membrane (Figure 4).

**Figure 5:**
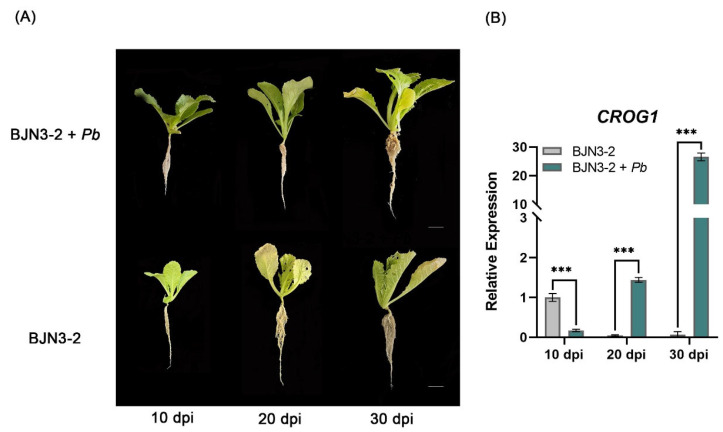
Disease investigation of Chinese cabbage inbred line BJN3-2 and BJN3-2 inoculated with *Plasmodiophora brassicae*. (**A**) Root phenotypes of Chinese cabbage inbred line BJN3-2 (*B. rapa*) inoculated with water or *P*. *brassicae* (*Pb*). Scale bar = 2 cm. (**B**) Validation of *CROG1* in BJN3-2 inoculated with water or *P*. *brassicae* (*Pb*) by RT-qPCR. The data are presented as means ± SE from three independent measurements. Black asterisks (***) indicate a significant difference (*t*-test, *p* < 0.05).

As *CROG1* is a unique orphan gene in the *Brassica* A genome, we analyzed its expression dynamics in *B*. *rapa* after *P*. *brassicae* infection. In the susceptible *B*. *rapa* inbred line BJN3-2, mock-inoculated plants showed no symptoms, whereas inoculated plants exhibited 100% disease incidence with severe root galling at 30 dpi (Figure 5A). *CROG1* transcript levels were measured at three time points after inoculation, revealing that *P*. *brassicae* infection significantly induced *CROG1* expression compared with the mock-inoculated control (Figure 5B), establishing that *CROG1* expression is responsive to *P*. *brassicae* inoculation.

### 2.4. Transcriptome Analysis of Arabidopsis thaliana CROG1 Transgenic Plants Inoculated with P. brassicae

To analyze the mechanism by which *CROG1* regulates clubroot resistance, root samples were collected from WT and *CROG1*-overexpressing (CROG1OE) plants at 3, 4, and 5 wpi for cDNA library construction and transcriptome sequencing. The sequencing quality was high, with the Q20 and Q30 base percentages of all of the samples exceeding 96.51% and 91.46%, respectively (Table S1). Alignment to the TAIR10 reference genome (http://www.arabidopsis.org) (accessed on 10 September 2024) was effective, yielding uniquely mapped read rates above 97.08% for all of the samples. These results confirm the reliability of the data for subsequent analyses (Table S2).

**Figure 6:**
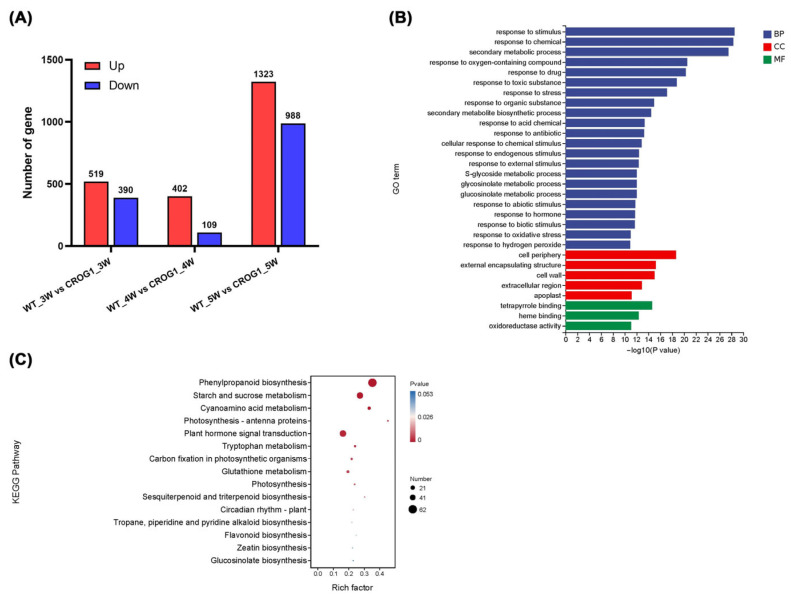
Identification of differentially expressed genes (DEGs) between wild-type (WT) and CROG1OE plants. Total RNA was isolated from root of *A. thaliana* Col-0 and *CROG1OE* plants at 3, 4, and 5 wpi, each with three biological replicates. (**A**) Number of DEGs. (**B**) DEG Gene Ontology (GO) classification. Individual bars denote the number of DEGs mapped to particular GO categories. Blue: biological process (BP), red: cellular component (CC), green: molecular function (MF). (**C**) KEGG pathways associated with the DEGs. Pathways are provided with the corresponding rich factors.

Transcriptome profiling revealed 3731 differentially expressed genes (DEGs) between WT and CROG1OE plants, with 2244 upregulated and 1487 downregulated DEGs (Figure 6A, Table S3). Gene Ontology (GO) analysis demonstrated significant enrichment of DEGs in key biological processes, including “response to stimulus” (GO:0050896) and “secondary metabolic process” (GO:0019748), and cellular components, including “cell wall” (GO:0005618) and “cell periphery” (GO:0071944) (Figure 6B). Kyoto Encyclopedia of Genes and Genomes (KEGG) pathway analysis identified three significantly enriched metabolic pathways: “phenylpropanoid biosynthesis” (ko00940), “starch and sucrose metabolism” (ko00500), and “plant hormone signal transduction” (ko04075) (Figure 6C). The phenylpropanoid biosynthesis pathway showed consistent enrichment across all three time point comparison groups. These findings suggest that *CROG1*-mediated clubroot resistance functions through the modulation of the phenylpropanoid biosynthesis pathway, having downstream effects on cell wall reinforcement and lignin deposition.

### 2.5. CROG1 Is Involved in Clubroot Resistance by Affecting the Phenylpropane Metabolic Pathway

**Figure 7:**
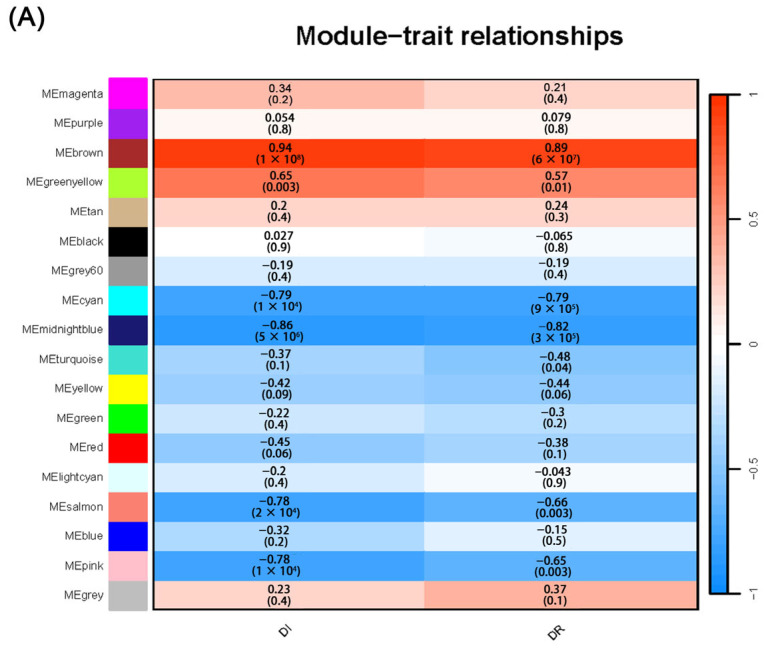

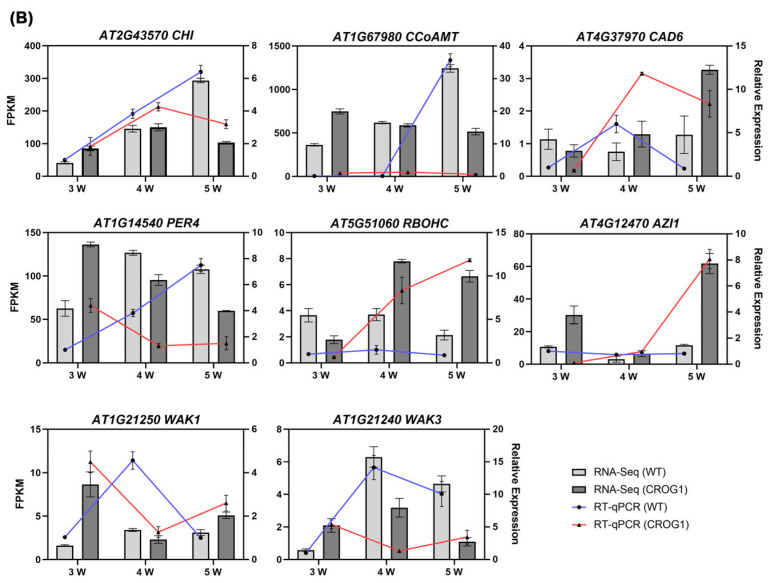
Transcriptome data, WGCNA, and RT-qPCR results. (**A**) Pearson correlation between modules and phenotypes. Red indicates a positive correlation; blue indicates a negative correlation; numbers in brackets represent *p*-values of correlations; outside brackets indicate correlation coefficients. (**B**) Validation of transcriptome data by RT-qPCR. *Arabidopsis thaliana* ecotype Col-0 (WT) and *CROG1* transgenic lines were observed 3–5 wpi with *Plasmodiophora brassicae*. The red solid line represents RT-qPCR for the CROG1 group; the blue solid line represents RT-qPCR for the WT group. The data are presented as means ± SE from three independent measurements. The grey bar chart represents RNA-seq expression for the *CROG1* group; the white bar chart represents RNA-seq expression for the WT group (one-way ANOVA, *p* < 0.05).

To investigate the molecular mechanisms underlying clubroot resistance, we integrated the DR and DI with the transcriptome data using weighted correlation network analysis (WGCNA). WGCNA identified 18 co-expression modules, among which the brown module showed the strongest positive correlation with disease phenotypes (Figure 7A). Functional enrichment analysis of genes within this module revealed that the phenylalanine, tyrosine, and tryptophan biosynthesis pathway was significantly enriched (Figure S4). These findings indicate that *CROG1* participates in clubroot resistance through its involvement in the phenylpropanoid metabolic pathway.

In the brown module, we identified genes involved in lignin synthesis and cell wall formation via the phenylpropanoid pathway, including *CCoAMT* and *CAD6*, which are established regulators of lignin biosynthesis [31]. Real-time quantitative polymerase chain reaction (RT-qPCR) validation confirmed that the expression patterns of these genes aligned with the transcriptome data, showing significant induction in CROG1OE plants compared with WT controls. In addition, *CHI* encodes a chitinase, a protein that plays important roles in plant defense against biotic and abiotic stressors. *CHI* expression differed significantly between CROG1OE and WT plants, suggesting that its expression may be regulated by *CROG1*.

Transcriptomic profiling revealed multiple DEGs functionally associated with plant defense responses, cell wall biosynthesis, and structural defense mechanisms. *PAL* is an entry enzyme for the phenylpropanoid pathway and is activated after sensing H_2_O_2_ accumulation. *Respiratory burst oxidase homolog* (*RBOHC)*, a marker gene of the plant defense response level, was significantly upregulated in CROG1OE plants, indicating that *CROG1* overexpression induces a more intense defense response in plants and may affect the phenylpropane metabolic pathway. *PER4* has been reported to play a role in cell elongation and plant–pathogen interactions. We found that the *PER4* transcript levels were affected by *CROG1* overexpression. AZI1, which improves plant disease resistance by enhancing lignin synthesis [32], was affected by *CROG1* overexpression and showed very high transcript levels at 5 wpi. In addition, cell wall-associated kinases showed a different trend in the *CROG1* overexpression lines compared with WT (Figure 7B). These transcriptional changes implicate the role of *CROG1* in modulating clubroot resistance through the regulation of the phenylpropanoid metabolic pathway and associated defense mechanisms.

## 3. Discussion

Orphan genes, which are uniquely present in plant genomes, remain understudied in current research and are largely confined to a limited number of species. Research has indicated their significant role not only in plant growth and development but in plant defense against biotic stress [33,34,35,36,37,38]. In cruciferous crops, the orphan gene *AtQQS* exemplifies this function and interacts with *AtNF-YC4* to enhance antiviral and antimicrobial immunity. *AtQQS* overexpression in *A*. *thaliana* and soybean increases plant resistance to aphids, fungal pathogens, and other microbes [34]. Studies have also shown that the cruciferous-specific orphan gene *EWR1* and its kale homolog *BoEWR1* confer resistance to Verticillium wilt [39]. Similarly, Jiang et al. [35] demonstrated that 41 of 52 assessed orphan genes (*BrOGs*) in *B*. *rapa* responded transcriptionally to *P*. *brassicae* infection. Collectively, these findings establish a strong association between orphan genes and plant biotic stress responses.

Liu et al. [2] demonstrated that *P. brassicae* infection is initiated in the host cortex and that cell wall modifications occur in both susceptible and resistant plant materials. The phenylpropanoid pathway, a crucial secondary metabolic process responsible for lignin and flavonoid synthesis, plays a significant role in biotic stress responses. Studies on *Brassica*–*P*. *brassicae* interactions have implicated the phenylpropanoid pathway and its branches. As *P*. *brassicae* lacks genes for cell wall degradation or modification [15], host-induced cell wall alterations during infection are likely derived from immune responses activated by disease resistance genes, such as *Rcr1* [24]. Transcriptomic analysis of *Rcr1*-mediated resistance revealed its potential influence on the jasmonic acid signaling pathway and the subsequent upregulation of phenylpropanoid pathway genes [20,40]. Tu et al. [16] observed enhanced lignification, particularly in the outer cortex of resistant plants, which impedes pathogen entry. This increase in lignification was attributed to the activation of genes involved in the phenylpropanoid pathway and the S-lignin unit biosynthesis branch. These findings collectively indicate that *Rcr1* confers resistance, at least partially, by inducing lignin accumulation. BJN3-2 is susceptible to clubroot, whereas *CROG1* remains upregulated following inoculation (Figure 5B). Moreover, overexpressing *CROG1* in *Arabidopsis* reduced the disease rate and disease index (Figure 1 and Figure 2), yet disease development was not completely prevented. Therefore, we propose that *CROG1* may contribute to resistance-related responses, but is not the only determining factor of resistance to clubroot. In our study, *CROG1* overexpression in *Arabidopsis* altered the expression of numerous genes, including several involved in phenylpropanoid metabolism, which exhibited differential expression patterns compared with WT plants after inoculation. Cell wall-associated kinases *WAK1* and *WAK3* have both been associated with plant antimicrobial responses [41,42], and their expression was affected in our experiments (Figure 6B). Therefore, we hypothesize that *CROG1* modulates clubroot resistance by influencing lignin or flavonoid biosynthesis via the phenylpropanoid pathway.

There are two theories that explain the origin of orphan genes. The first posits that they arose through rapid sequence evolution and lost detectable homology to other genes over evolutionary time [43]. The second theory suggests that de novo evolution occurred in non-coding genomic regions, offering a framework for understanding their functional emergence [44,45,46]. Compared with evolutionarily conserved genes, orphan genes exhibit accelerated evolutionary rates and high sequence specificity, resulting in strong species specificity. *Plasmodiophora brassicae* exhibits host specificity for cruciferous crops, and there are several characterized disease resistance loci in the *B*. *rapa* genome. Stressors induce genomic changes, including gene birth and death events [47]. It remains unknown whether selective pressure from *P*. *brassicae* infection drives the evolution of orphan genes in *B*. *rapa*. Supporting this hypothesis, our study showed that overexpression of the orphan gene *CROG1* reduced clubroot resistance in *A*. *thaliana* (Figure 2). Furthermore, *CROG1* expression was significantly induced by *P*. *brassicae* in susceptible Chinese cabbage (*B*. *rapa*) lines (Figure 3). Collectively, these findings suggest that the selective pressures imposed by *P*. *brassicae* during the evolution of *B*. *rapa* could have generated orphan genes associated with susceptibility or resistance mechanisms.

Here, we report *CROG1* as an orphan gene associated with clubroot resistance. Transcriptome analysis suggests that *CROG1* influences resistance, potentially by modulating lignin biosynthesis and/or cell wall integrity. However, this is a preliminary finding, and further molecular studies are needed to determine how *CROG1* integrates into the complex signaling networks governing the host–*P*. *brassicae* interaction. Our results provide new avenues for enhancing disease resistance in cruciferous crops.

## 4. Materials and Methods

### 4.1. Plant Materials

*A. thaliana* ecotype Col-0 and 20 BrOG transgenic lines [30] were used to screen plant materials responding to *Plasmodiophora brassicae*. They were grown under long day conditions (16/8 h light/dark photoperiod) at 22 ± 1 °C with a cool-white fluorescent light and a relative humidity of 65–70%. *Brassica rapa* inbred line ‘BJN3-2’ is susceptible to clubroot disease [21]. It was inoculated with *P*. *brassicae* to determine the expression pattern of *CROG1* in *B*. *rapa*.

### 4.2. Plasmodiophora brassicae Inoculation

The pathotype used for *P*. *brassicae* identification was ‘LAB3’, which was identified as Pb4 by the SCD system [48]. The roots were ground with water, filtered with gauze, and collected in a 50 mL centrifuge tube. The spore count of the filtered bacterial solution was determined using a hemocytometer. The spore concentration was adjusted to 10^7^ spores/mL. We selected 4-week-old *A*. *thaliana* plants and injected spore solution into their roots. *Brassica rapa* inbred line BJN3-2 was inoculated at the 2–3 true leaf stage. Each plant was injected with 2 mL of spore solution into the roots [26,49]. Each treatment was conducted with three biological replicates, each consisting of at least twelve individual plants.

### 4.3. Clubroot Disease Assessment

The disease rate (DR) and disease index (DI) were determined in *A*. *thaliana* at 3, 4, and 5 wpi with *P*. *brassicae*. Disease severity was divided into five levels: level 0, asymptomatic; level 1, small galls on lateral roots; level 2, larger galls on lateral roots; level 3, galls on the main root; and level 4, galls on both main and lateral roots. DR was calculated by dividing the number of non-0 level plants by the total number of inoculated plants. DI was calculated according to the following formula:

DI = [(*n_1_* + 2*n_2_* + 3*n_3_* + 4*n_4_*)/*N_T_*× 4] × 100,

where *n_i_* is the number of plants in each disease severity class, and *N_T_* is the total number of plants tested [50]. Total root DNA was extracted using the cetyltrimethylammonium bromide (CTAB) method, and the DNA content of *P*. *brassicae* was quantified using specific primers, named Pb-F, Pb-R, Fbox-F, and Fbox-R [26], the sequences of which are shown in Table S4.

### 4.4. Inoculation with Pseudomonas syringae pv. Tomato DC3000

Four-week-old *A*. *thaliana* wild type and transgenic lines BrOG36OE, BrOG51OE, and BrOG103OE were hand-infiltrated with a *Pseudomonas syringae pv. tomato DC3000 (Pst DC3000)* bacterial suspension (OD_600_ = 0.0002 in 10 mM MgCl_2_), and the bacterial load was quantified at 3 dpi. *Ps*. *syringae* pv. *tomato* DC3000 was cultured as previously described 50. There were three biological replicates.

### 4.5. RNA Extraction and RT-qPCR Analysis

Total RNA was isolated from the root of Col-0 and *BrOGOE* plants at 3, 4, and 5 wpi, and BJN3-2 at 10, 20, and 30 dpi using the TRNzol universal reagent (4992730; TIANGEN; Beijing, China), each with three biological replicates. Key genes were detected using RT-qPCR. cDNA was synthesized from total RNA using a PrimeScript RT Reagent Kit with gDNA Eraser (Perfect Real Time; RR047A; TaKaRa; China) according to the manufacturer’s instructions. Relative expression was calculated using the 2^−ΔΔCT^ method. Each assay was repeated three times. The primer sequences for RT-qPCR are listed in Table S4.

### 4.6. Subcellular Localization Assays

To determine the subcellular localization of CROG1, a fusion construct was generated by cloning the full-length coding sequence of *CROG1* (*BraA05004304*) into the pCAMBIA1302-GFP vector downstream of the Cauliflower Mosaic Virus (CaMV) 35S constitutive promoter, creating a 35S::CROG1::GFP translational fusion. The empty pCAMBIA1302-GFP vector (35S::GFP) served as a control. *Agrobacterium tumefaciens* strain GV3101 harboring either the fusion construct or the control vector was individually infiltrated into the abaxial air spaces of young leaves of 4–5-week-old *Nicotiana benthamiana* plants using the transient agroinfiltration method. Infiltrated plants were maintained under standard growth conditions (22 °C, 16 h light/8 h dark cycle) for 48–72 h post-infiltration to allow optimal protein expression. They were examined across three biological replicates.

### 4.7. Library Construction and RNA-Seq

Library construction and sequencing were performed by Shanghai Personalbio Technology Co., Ltd. (Shanghai, China). Total RNA was isolated from root of Col-0 and *CROG1OE* plants at 3 wpi, 4 wpi, and 5 wpi after infection, each with three biological replicates. Firstly, mRNA was purified from total RNA using poly-T oligo-attached magnetic beads. First-strand cDNA was synthesized using random oligonucleotides and Super Script II. Second-strand cDNA synthesis was subsequently performed using DNA Polymerase I and RNase H. Remaining overhangs were converted into blunt ends via exonuclease/polymerase activities, and the enzymes were removed. The library fragments were purified using the AMPure XP system (Beckman Coulter, Beverly, CA, USA). The sequencing library was then sequenced on the NovaSeq 6000 platform (Illumina, Shanghai, China).

### 4.8. RNA-Seq Data Analysis

After sequencing, the reads with low sequencing quality or sequencing connectors in the raw data were filtered. The *Arabidopsis* genome (TAIR10) was used as the reference genome (http://www.arabidopsis.org) (accessed on 10 September 2024). The filtered reads were mapped to the reference genome using HISAT2 (v2.1.0). For each transcription region, the fragment per kilobase of transcript per million mapped reads (FPKM) value was calculated to quantify its expression abundance and variations using RSEM software (v1.2.31). Then, the difference expression of genes was analyzed by DESeq (v1.38.3) with screened conditions as follows: expression difference multiple |log_2_FoldChange| > 1. The DEGs were then subjected to KEGG enrichment analysis and GO enrichment analysis.

### 4.9. Weighted Gene Co-Expression Network Analysis (WGCNA)

WGCNA was performed to identify functionally coordinated gene modules associated with *CROG1*-mediated clubroot resistance, using high-quality transcriptome data from root tissues of Col-0 (WT) and CROG1OE *Arabidopsis* plants at 3, 4, and 5 wpi. Weighted gene co-expression networks were generated in R (version 3.4.2) utilizing the WGCNA package, employing its automated network construction function (blockwiseModules) with default parameters to identify gene modules. Key modules showing strong genotype-dependent enrichment (*p* < 10^−5^) were functionally annotated via Gene Ontology (GO) and Kyoto Encyclopedia of Genes and Genomes (KEGG) analyses.

### 4.10. Statistical Analysis

Data were analyzed with SPSS v19.0 software using Student’s *t*-test or one-way ANOVA followed by individual comparisons with Tukey’s test, significant *p*-value < 0.05.

## 5. Conclusions

Based on the functional screening of a *Brassica rapa* orphan gene (BrOG) overexpression library in *Arabidopsis thaliana*, this study identifies *BrOG51* (designated *CROG1*) as a novel *B. rapa*-specific orphan gene conferring significant resistance to *Plasmodiophora brassicae*. Overexpression of *CROG1* in *Arabidopsis* substantially reduced disease incidence and severity, suppressed pathogen proliferation, and was transcriptionally induced in susceptible *B. rapa* upon pathogen challenge. Transcriptome profiling and weighted gene co-expression network analysis (WGCNA) revealed that *CROG1* orchestrates clubroot resistance primarily by modulating the phenylpropanoid biosynthesis pathway, leading to the upregulation of key lignin synthesis genes (*CCoAMT*, *CAD6*, *PER4*, *AZI1*) and defense regulators (*RBOHC*, *WAKs*), thereby enhancing cell wall fortification through lignin deposition and structural modifications. These findings provide the first evidence of a *Brassica*-specific orphan gene directly regulating clubroot resistance via phenylpropanoid-mediated defense mechanisms, offering a novel genetic target and molecular strategy for breeding resistant *Brassica* crops.

## Data Availability

All data supporting the findings of this study are available within the paper and within its Supplementary Materials published online.

## Supplementary Materials

The following supporting information can be downloaded at: https://www.mdpi.com/article/10.3390/plants14172683/s1, Figure S1: Phenotypic observation of Arabidopsis thaliana WT and three BrOGOE transgenic lines (BrOG36OE, BrOG51OE, and BrOG103OE) at 4 wpi with Plasmodiophora brassicae. WT represents A. thaliana ecotype Col-0; Figure S2: Leaf phenotype, disease symptom, and AtPR1 gene expression level in Arabidopsis thaliana WT and three BrOGOE transgenic lines (BrOG36OE, BrOG51OE, and BrOG103OE) at 3 dpi with Pseudomonas syringae pv. tomato DC3000. WT represents A. thaliana ecotype Col-0; Figure S3: Gene sequence and amino acid sequence of CROG1 (BraA05004304). The CROG1 gene contains an intron spanning from 111 bp to 697 bp, flanked by exonic regions from bp 1 to 110 bp and 698 to 737 bp; Figure S4: KEGG and GO analysis of genes in the brown module of WGCNA. Table S1: Transcriptome sequencing quality data; Table S2: Summary of transcriptome data map situation; Table S3: Differentially expressed gene information; Table S4: Primer sequences used in this study.

## Abbreviations

The following abbreviations are used in this manuscript:

*OGs*	*Orphan genes*
*CROG1*	*Clubroot-related orphan gene 1*
*CCoAMT*	*Transcaffeoyl Coenzyme A 3-O-methyltransferase*
*CAD6*	*Cinnamyl alcohol dehydrogenase 6*
*PER4*	*PEROXIDASE 4*
*AZI1*	*AZELAIC ACID INDUCED 1*
*RBOHC*	*RESPIRATORY BURST OXIDASE HOMOLOG C*
*WAKS*	*WALL-ASSOCIATED KINASES*
*EGT*	*Ergothioneine*
*DR*	*Disease rating*
*DI*	*Disease index*
*OE*	*Overexpression*
*dpi*	*Days post-inoculation*
*wpi*	*Weeks post-inoculation*
*WT*	*Wild-type*
*RT-qPCR*	*Quantitative real-time polymerase chain reaction*
*GO*	*Gene Ontology*
*DEGs*	*Differentially expressed genes*
*WGCNA*	*Weighted correlation network analysis*
*AtQQS*	*Arabidopsis Qua-Quine Starch*
*FPKM*	*Fragments per kilobase of transcript per million mapped*
