# Supplementary figures and images for: Brassica-Specific Orphan Gene CROG1 Confers Clubroot Resistance in Arabidopsis via Phenylpropanoid Pathway Activation
Source: Plants (Basel). 2025 Aug 27;14(17):2683. doi: 10.3390/plants14172683 (PMC12430220; doi:10.3390/plants14172683)

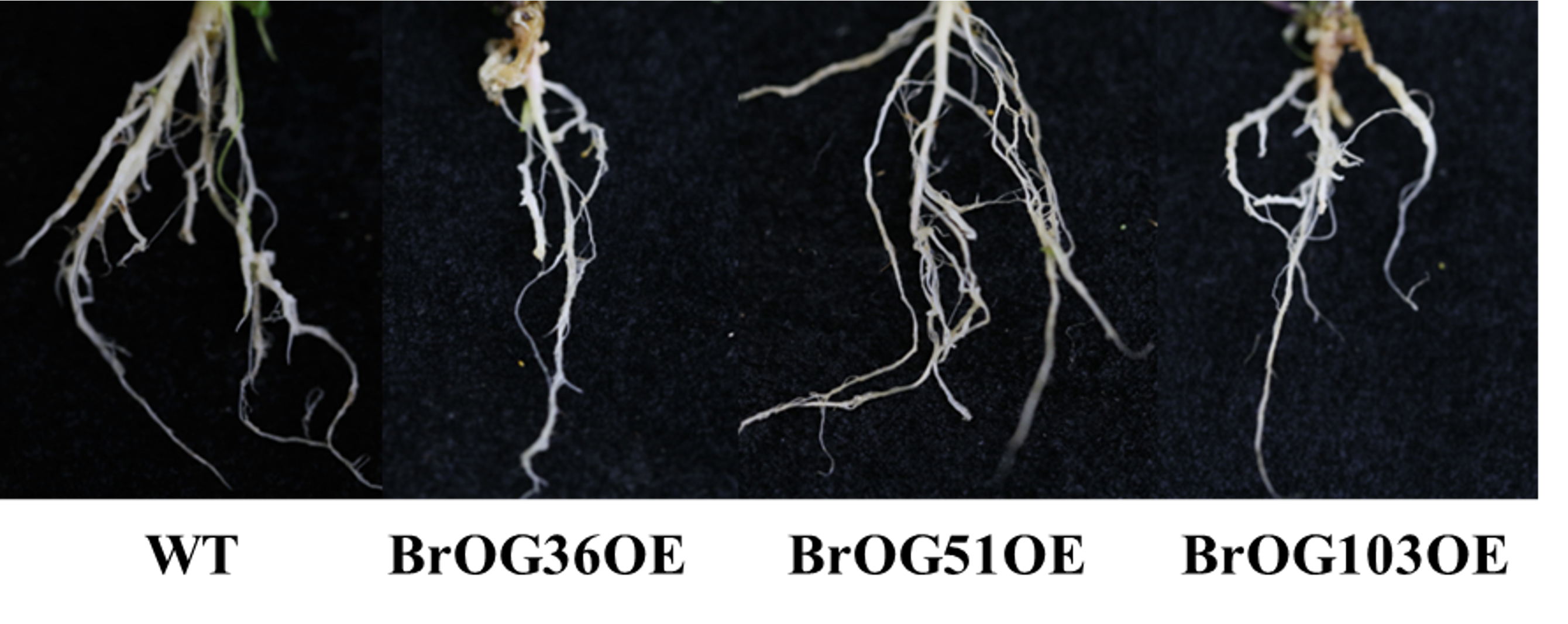

Supplement: Supplementary file 1 [file plants-14-02683-s001.zip › Figure S1.tif]

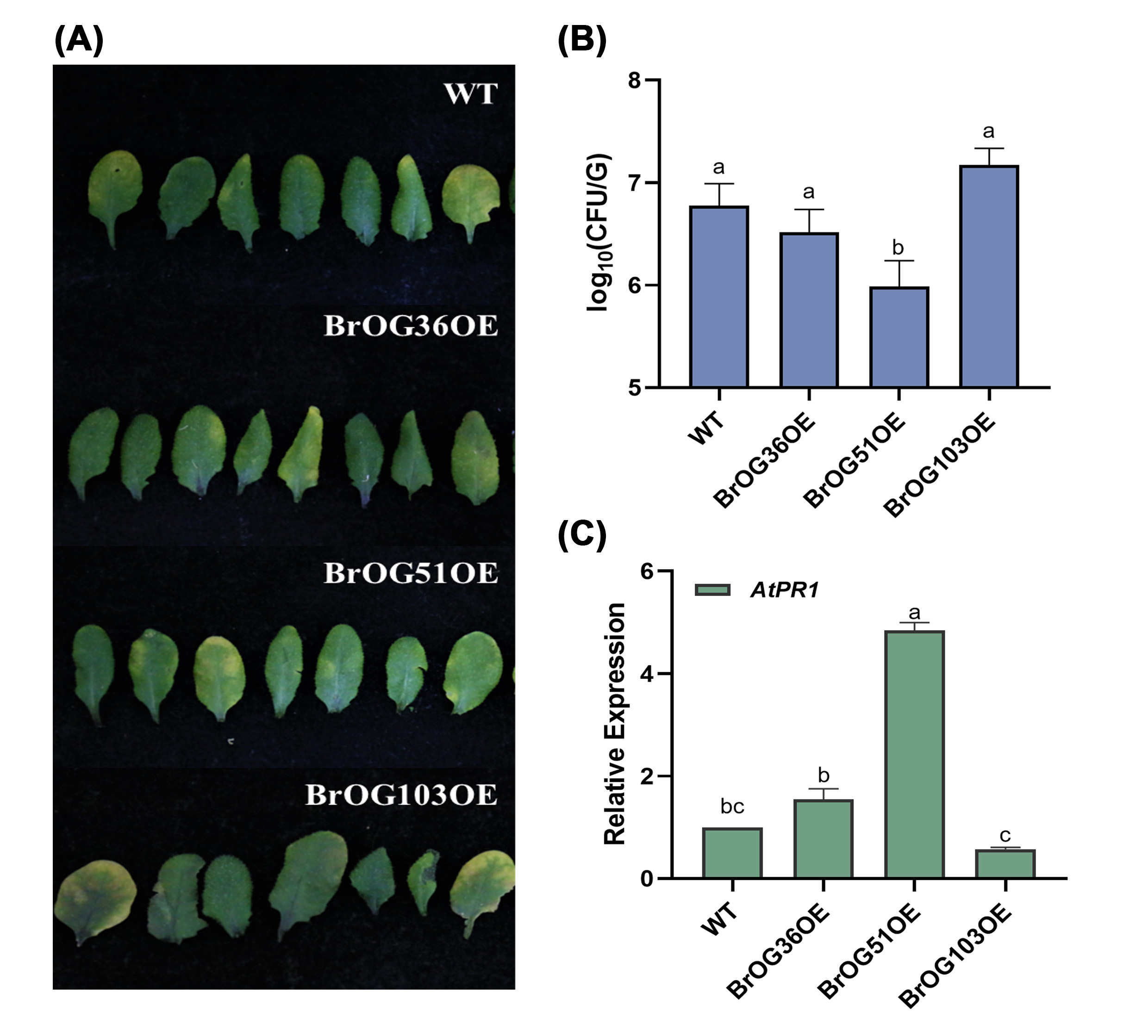

Supplement: Supplementary file 1 [file plants-14-02683-s001.zip › Figure S2.jpg]

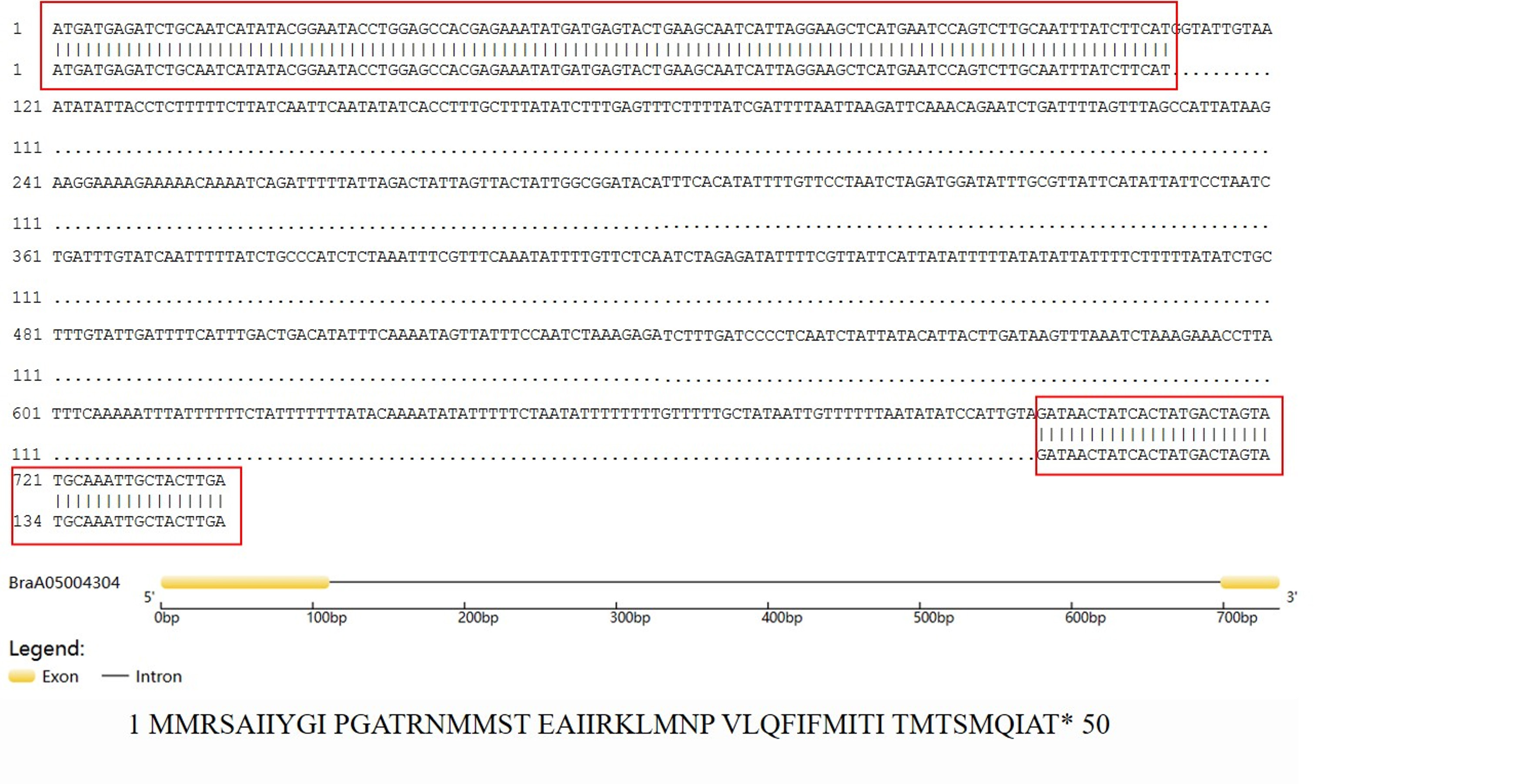

Supplement: Supplementary file 1 [file plants-14-02683-s001.zip › Figure S3.jpg]

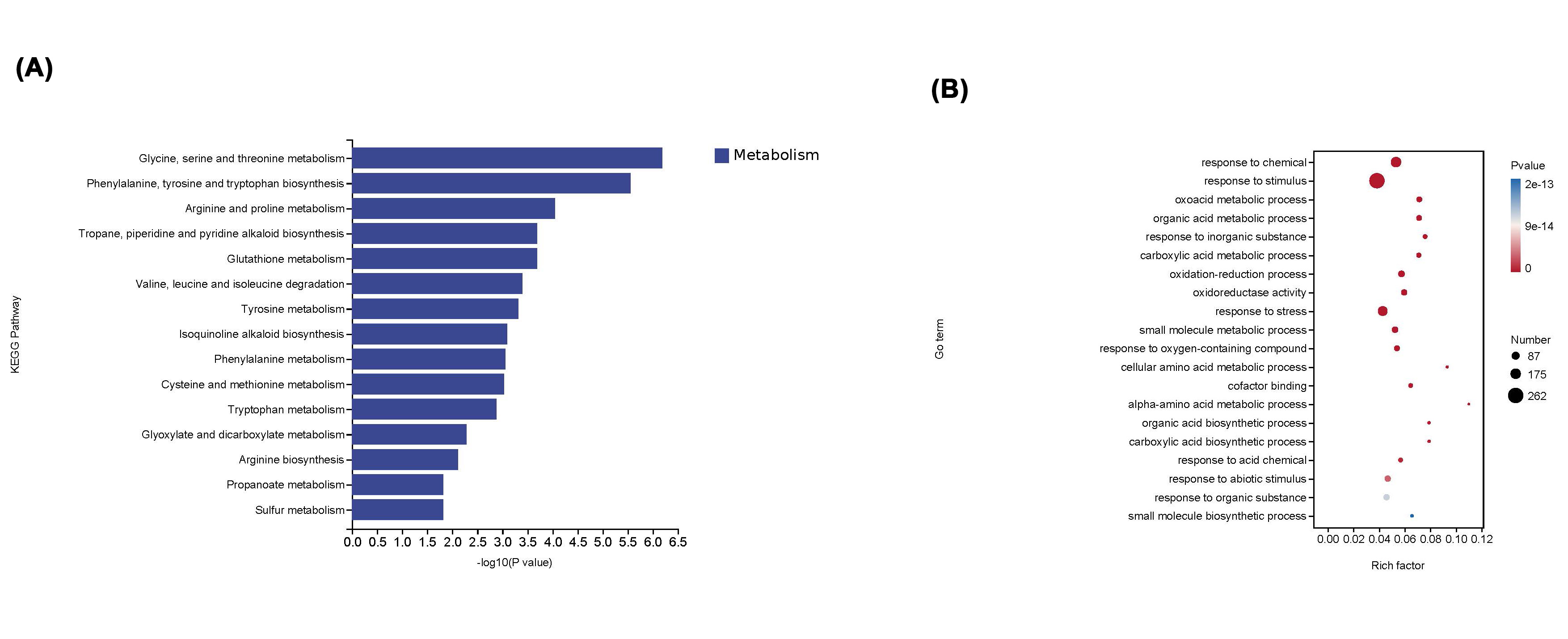

Supplement: Supplementary file 1 [file plants-14-02683-s001.zip › Figure S4.jpg]
